# Different Points of a Continuum? Cross Sectional Comparison of the Current and Pre-contact Psychosocial Problems among the Different Categories of Adolescents in Institutional Care in Nigeria

**DOI:** 10.1186/1471-2458-12-554

**Published:** 2012-07-26

**Authors:** Olayinka Atilola

**Affiliations:** 1 Dept of Psychiatry, University College Hospital Ibadan, Ibadan, Nigeria

**Keywords:** Juvenile justice, Social welfare, Custodial care, Juvenile delinquency, Child neglect

## Abstract

**Background:**

The combination of adverse social indicators and a predominantly youthful population puts Nigeria, and indeed many countries of sub-Sahara Africa, at the risk of explosion in the number of youth coming in contact with the juvenile justice system. Despite this risk, custodial childcare systems in the region are still poorly developed with both juvenile offenders and neglected adolescents coming in contact with the systems being kept in the same incarcerating facility. The needs of these different groups of adolescents may be different. Knowing their common and unique needs can inform common prevention strategies and ensure that specific service-needs of different categories of adolescents in institutional custody are met.

**Methods:**

Data on the family background, pre-contact social circumstance, neurological and anthropometric profiles, and certain aspects of mental health of adolescents drawn from two juvenile justice institutions in Nigeria were obtained. The results for the adolescents on ‘criminal code’ and those admitted as a case of child neglect were compared using chi-square and odd ratios.

**Results:**

Participants were 211 adolescents comprising of 158 on ‘criminal code’ and 53 declared as ‘neglected’. A lot of similarities were found. For instance, the prevalence of parental separation, family transition, experience of street-life and lifetime exposure to traumatic events and posttraumatic stress was equally high among the two groups of adolescents. The adolescents on ‘criminal code’ however had significantly higher prevalence of conduct and alcohol/substance use disorders while the child neglect group had poorer anthropometric profiles and higher prevalence of neurological disorders.

**Conclusions:**

Child neglect and juvenile delinquency in Nigeria may truly be different points of a continuum. There are however fundamental differences that can warrant specific prevention strategies and tailor-made service provision while in custodial care.

## Background

The ongoing global economic crisis and its crippling effect on resources for qualitative livelihood, is bound to alter the care environment of children and adolescents. Among the reported implications of the on-going economic meltdown is a global rise in cases of child maltreatment and neglect [1], as well as juvenile delinquency and youth crime [2]. The number of children with such adverse social outcomes is even bound to be higher in the developing and resource poor countries. This is because these countries which harbour up to 85% of world’s child and adolescent population [3] are also the worst-hit by the ongoing economic crisis [4]. Sub-Sahara Africa in particular presents a situation for an explosion in the cases of child neglect as well as juvenile delinquency, in view of the particularly high child and adolescent population, worsening poverty, widening social inequality and rising unemployment in the region [5,6].

Studies from different parts of sub-Sahara Africa have reported rising cases of juvenile justice contact among adolescents. Maru et al. in 2003 reported a rise of about 21% in the number of adolescents coming to juvenile justice courts in Nairobi Kenya [7]. A situation analysis conducted by UNICEF in Uganda also established similar trends of increase in Kampala city [8]. The comptroller of Nigerian prisons also reported that the number of adolescents processed in the juvenile justice wing of the Prison Services has tripled between the year 2008 and 2011[9]. Life of want, deprivation, abuse and neglect has also led a lot of children and adolescents in the region to take to the streets as destitute [2,5,10], many of whom also invariably come in contact with the juvenile justice system. This is not unconnected with the fact that subsistence on the street often times requires children to recourse to crime [11,12] and in many jurisdictions in the region being a street-child itself is a status offence. Assiago in 2002 reported that a large proportion of adolescents in Kenyan juvenile justice institutions were street children who had not broken any law beyond being homeless [13].

Despite the apparently high burden of juvenile delinquency and need for child and adolescent social welfare services in many parts of sub-Saharan Africa, the juvenile justice and social welfare systems in these regions are still poorly developed [14,15]. Childcare services in many of sub-Saharan African countries utilise mostly incarcerating methods of custodial care. Also, criminalisation of child destitution in many sub-Saharan African countries entails that there is no clear distinction between juvenile crime or delinquency and youth abandonment or destitution. In a study in Kenya, adolescents who were found destitute were among the adolescents brought before a district juvenile court for ‘sentencing’ to remand homes [7]. Okumu in 2007 reported that maltreated or neglected adolescents as well as juvenile offenders were in about equal proportions among the ‘inmates’ of the Getathuru Children's Rehabilitation Centre in Nairobi Kenya [16]. Similarly in Nigeria, Bella et al. in 2010 reported that neglected adolescents in need of care and protection as well as status offenders, young minor offenders and adolescents declared as ‘beyond parental control’ were processed through the same legal framework and remanded together in the same confinement [17]. This scenario has created a situation whereby being a victim of child neglect became criminalised with diminished chances of attending to the peculiar needs of such adolescents. The scenario also created a situation of lack of clear distinction in the programmes and schemes for the care, reformation, rehabilitation and re-integration of the different categories of children and adolescents in childcare custodial services. The result of this is that current prevention strategies and service provision for children and adolescents in custodial care in sub-Sahara Africa, where it exists at all, assume that the needs and challenges of the inmates are the same irrespective of their entry pathways.

In line with this assumption, previous studies on predisposing factors and psychosocial needs-assessments of children and adolescents within the juvenile justice system in sub-Sahara Africa, though very few, had arbitrarily viewed them as one and the same without cognizance of their different reasons for contact [7,16-19]. These studies presuppose that the predisposing factors and the psycho-social needs of children and adolescents within the juvenile justice system are the same irrespective of their different pathways into the system. They also made blanket recommendations on prevention strategies and service provisioning for all categories of adolescents in custodial care based on these assumptions. While it is a common and safe assumption that most children in difficult circumstances in sub-Sahara Africa share a lot in common in terms of vulnerabilities and psychosocial needs [6,13], these assumptions has not been put to objective test in the context of adolescents in the juvenile justice system.

There is no doubt that any pre-emptive or preventive strategies towards, or any form of service provisioning framework on the issue of children and adolescents coming in contact with the juvenile justice system in sub-Sahara Africa are worthwhile. However, rather than continuing the practice of viewing all the different categories of children and adolescents coming in contact with the juvenile justice system in this region as one and the same, identifying the fundamental similarities and differences in their social, physical and psychological needs will provide a framework for a better focussed strategy. Such information can serve as a more reliable template for common and specific targets in large scale prevention programmes. Aside this, understanding the unique and distinct psychosocial needs of children and adolescents within the juvenile justice and social welfare systems can, and has been found to, facilitate the provision of the appropriate services for them [20,21]. Provision of needed psychosocial and rehabilitative services to children and adolescents in institutional custody will improve their quality of life, enhance their societal re-integration and reduce the risk of recidivism.

Among the countries in sub-Sahara Africa, Nigeria is of particular interest in terms of the risk of exponential rise in the number of children and adolescents coming in contact with the juvenile justice system. Other than having a predominantly youthful population; Nigeria accounts for almost a quarter of the entire child and adolescent population of sub-Sahara Africa [22]. Aside this, the country has one of the highest rate of household poverty and among the countries with the lowest Human Development Index in the region [23]. Expectedly, recent reports suggest that the burden of children and adolescents coming or at risk of coming in contact with the social-welfare and juvenile justice systems in Nigeria is rising [9]. There are also reports that the streets of Nigeria are being inundated daily with hordes of different categories of neglected children and adolescents [24,25] who are potential entrants into the juvenile justice system. Just like in many countries of sub-Sahara Africa, there is a lack of clear distinction between the criminal and social welfare aspects of childcare services in Nigeria. Neglected adolescents, adolescents adjudged as delinquent, as well as young offenders in the country are all processed through the same system and can be remanded together in the same facility [26].

The present study therefore examined the similarities and differences in the pre-contact family structure and functioning; pre-contact social circumstance, and the current physical and mental status between adolescents on ‘criminal code’ and those declared as neglected in two different types of juvenile institutions in Nigeria. It was assumed that the similarities and differences between these two groups of adolescents in terms of their pre-contact family and social situation can shed some light on the common and specific pathways into the juvenile justice and social welfare systems among adolescents in institutional care in Nigeria. Such findings can help in the design of common and specific prevention programmes. The similarities and differences in the current physical and mental status of the two categories of adolescents may also mirror the common as well as specific physical and psycho-social needs of these adolescents while in institutional care. Such findings may point the direction for the common and specific psychosocial interventions necessary for successful rehabilitation and re-integration of such children and adolescents.

## Methods

### Study setting

Being the two types of institutional care for children and adolescents in the country, a convenient sample of one of the 3 borstal homes and one of the 24 remand homes in Nigeria were selected for this study. The selected facilities included the Abeokuta Juvenile Borstal Home and the Ibadan Juvenile Remand Home, both in South-west Nigeria. Borstal homes in Nigeria are under the control of the central government of Nigeria through the Nigerian Prison Services. They serve as a temporary detention centre for male adolescents between the age of 16 and 20 years who are being remanded on ‘criminal code’ either as juvenile offenders or as adolescents declared as beyond parental control. On the other hand, remand homes in Nigeria are under the control of the state governments through their social welfare establishments. They serve as a multipurpose institution where abused/neglected children aged between 6 and 18 years and those on ‘criminal code’ are temporarily kept together for care, protection and reformation.

### Study instruments

Socio-demographic and family background questionnaire: This questionnaire was designed by the author and it has two sections. The first section of this questionnaire enquires about basic demographics as well as information on reason for admission in the home and length of stay so far**.** The second section enquires about family background and social circumstance before coming into the home. The domains of the family unit examined were family structure, family transition, and indices of family stability. These domains have been found to have content validity in the context of the role of the family in child outcomes [27,28].

Neurological and anthropometric evaluation pro-forma: This was developed with the assistance of a paediatric neurologist and was in the form of a pre-designed pro-forma. Columns were created in which neurological findings were recorded. Neurological examinations included assessment of all the cranial nerves, cerebellar functions as well as tone and reflexes in the four limbs were done for all participants and recorded in a pre-designed pro-forma. History of epilepsy (including eye-witness accounts) was also obtained. Anthropometric profiles of the participants including weight and height as well as computed body mass index were also obtained and recorded.

Kiddies schedule for affective disorders and schizophrenia (KSADS): This is a is a semi-structured diagnostic interview designed to assess psychopathology in children and adolescents in accordance with the fourth edition of the Diagnostic and Statistical Manual (DSM-IV) criteria. This was used to assess for lifetime prevalence of those mental health problems which are known to be particularly common among adolescents within the juvenile justice and social welfare institutions. These included disruptive behaviour disorders [7,29-31], alcohol and substance abuse [18,32], lifetime exposure to multiple traumatic events [33,34] and posttraumatic stress disorder [18,33].

### Procedures

All adolescents aged between 10 – 20 years that were resident in the institutions were sampled. The cut-off point of 10–20 years was in line with the World Health organisation’s definition of adolescents as persons between the age of 10 and 19 years [35]. This translated to a total of 144 residents of the Abeokuta Borstal Home and 67 adolescents in the Ibadan Remand Home. Adolescents with severe neurological or obvious intellectual disability which made comprehension of the inquiries contained in the study questionnaire difficult were excluded from the interview aspects of the evaluation. Evaluation of their neurological and anthropometric profiles was however done. All interviews were conducted in English or Yoruba languages (depending on participant’s choice and adjudged fluency) in a face-to-face interview, ensuring that the adolescents had enough privacy. The Yoruba version of the socio-demographic questionnaire and the KSADS were generated by the ‘Translation and Back-translation’ method between a psychiatrist and a Yoruba-linguist until agreement was reached on its literary accuracy. The neurological examination and the interviews were conducted by the author. All physical examinations were done with minimal bodily exposure and conducted in a screened corner.

### Ethical considerations

Ethical permission to conduct the study was obtained from the Ethical Committee of the Neuropsychiatric Hospital Aro, Abeokuta and the Ethical Committee of the Oyo state Ministry of Health. Approval to interview residents of the Abeokuta Borstal Home and the Ibadan Remand Home was sought from the Ogun state command of the Nigerian Prison Services, and the Oyo State Ministry of Women Affairs, Social Welfare and Community Development respectively. In view of the difficulties likely to be involved in tracing parents and competent guardians of the participants, individual assent/consent was obtained from all participants. This procedure had been taken to be adequate in such circumstance especially in low risk research like this one [36,37].

### Data handling

The data collected were analyzed using the Statistical Package for Social Sciences version 16 (SPSS – 16) soft ware. Analysis was limited to bivariate descriptive statistics. The similarities and difference in some indices of family background, pre-contact social circumstances, and current and lifetime physical/psycho-social needs of the two categories of adolescents in the Homes were recorded. Similarities and significant differences (p < 0.05) in terms of unfavourable indices were taken as an indicator of common and specific factors that could have contributed to the current social outcomes of the adolescents. Same was also taken as an indicator of their common and specific psychosocial needs for well-being and successful rehabilitation while in the Homes.

## Results

A total of 211 adolescents comprising 158 adolescents on ‘criminal code’ and 53 adolescents admitted for care and protection (as a result of maltreatment or neglect) participated in the study. While all the adolescents on ‘criminal code’ sampled were fully included in the study, 7 of the neglected adolescents were excluded from the interview part of the evaluation on the grounds of gross neurological and subjective intellectual impairments that was adjudged likely to impede full comprehension of the interview.

### Socio-demographic characteristics and family background

Socio-demographics: The mean age of the participants was 15.8 ± 2.1 but the ‘criminal code’ participants were much older than the neglected adolescents (17.5 years ± 1.5 vs. 12.5 years ± 2.1; t = 6.5, p = 0.001). There were 185 boys (87.6%) in total with the ‘criminal code’ group having a comparatively higher proportion of boys (96% vs. 74%, χ ^2^ 22.0, p = 0.03). The reasons for placement of the adolescents in their respective categories and by extension, in the home are as shown in Table 1. There was no statistically significant difference in the length of time that the two groups had spent in confinement.

**Table 1:** Categories of adolescents in custodial institutions and reasons for their placement

**‘Criminal Code**	**Care and Protection**
Beyond parental control’ (83; 52.5%)	Picked up by law enforcement agents haven been found abandoned, homeless or wandering the streets (39; 86.8%)
Theft (61; 38.6%)	
Membership of and involvement in activities of armed gangs (8; 5.0%)	Found engaged in dangerous child labour like open-bus conducting (6; 11.3%)
Sexual offenses (4; 2.5%)	Adolescent girl living in a brothel (1; 1.9%)
Vandalism (2; 1.2%).	**Total: 53 (100%)**
**Total: 158 (100%)**	

Family background: The two groups shared a lot in common in terms of problematic indices of family structure as shown in Table 2. These similarities included a high prevalence (up to 60%) of parental separation among the two groups of participants. Changing family structure (family transitions) is another common family problem. For instance about a third of the two groups had lived with at least one other carer other than their parents and about half of their mothers had changed marital partner at least once in participants’ lifetime. Family transitions as a common problematic family experience among the participants was further buttressed by the sharp drop in the rates of parental involvement in the early-childhood care-giving and the immediate pre-contact care-giving in the two groups. For instance as shown in Table 2, about 56% of the adolescents on ‘criminal code’ were reportedly raised by parents, only 36% of them reported living with at least a parent as at the time of juvenile justice contact. The drop rate for the neglected adolescents was also sharp, from 36% to 17%.

**Table 2:** Similarity and differences in indices of static family structure between the two categories of adolescents in institutional care in Nigeria

**Variable**	**Adolescent on Criminal Code (N = 158)**	**Cases of Child/Adolescent Neglect (N = 46)**	**OR (95% CI)**	**χ**^**2**^	**p**
Family type					
Monogamous Vs. Polygamous	92(58)	22 (48)	1.50 (0.78 -2.94)	1.5	0.21
Parental marital status					
Single/separated/widowed Vs. Married/cohabiting	94 (60)	30 (65)	1.27 (0.67 -2.5.3)	0.5	0.48
Occupation of father					
Elementary occupation/unemployed Vs. Employed	100 (63.3)	18 (39.1)	2.68 (1.36 – 5.26)	8.5	**0.003**
Occupation of mother					
Elementary occupation/unemployed Vs. Employed	39 (24.7)	34(73.9)	0.11 (0.05 -0.24)	37.6	**<0.001**
Total number of mother’s children					
< 4 Vs. ≥ 4	88 (54)	22 (48)	0.26 (0.11 –1.60)	10.9	0.66
Total number of father’s children					
≤ 4 Vs. > 4	64 (41)	26 (57)	0.52 (0.27 – 1.01)	3.7	0.54

The severity of family transitions was however significantly higher among the neglected adolescents as they had a higher mean number of different care-givers at different points in their lives and their mothers also cohabited with a significantly higher mean number of partners (Table 3). Events that suggests family distress or instability like parental alcohol and substance abuse, domestic fights among parents and child physical abuse were quite high among the two groups, but the neglected adolescents were about 8 times more likely to have been witness to domestic fights at home than the adolescents on ‘criminal code’ (Table 3). Also, the social circumstance of the two groups of participants before their contact with the juvenile justice system was grim, as at least two-third of each group had dropped out of school and had been living on the streets for some time. However, the child neglect cases were almost twice as likely to have dropped out of school. Those on ‘criminal code’ on the other hand had been living on the streets for a higher mean number of months before contact (Table 4).

**Table 3:** Similarity and differences in indices of family consistency and stability between the two categories of adolescents in institutional care in Nigeria

**Variable**	**Adolescents on Criminal Code (N = 158)**	**Cases of Child/Adolescent Neglect (N = 46)**	**OR (95% CI)**	**χ**^**2**^	**p**
Current care-giver*					
At least one parent involved Vs. No parent involved	53 (34)	8 (17)	2.3 (1.04 – 5.55)	4.4	**0.03**
Early childhood care-giver					
Both parents Vs. Others	88 (56)	20 (36)	1.7 (1.16 - 4.45)	5.3	**0.02**
Lifetime number of different people that participant had lived with (other than parents)					
None Vs. At least one	62(39)	12 (26)	1.8 (0.88 – 3.80)	2.8	0.07
Mean number of different people that participant had lived with	3.5 ± 0.5	4.8 ± 0.3	--------------	7.7**	**0.002**
Lifetime number of persons that mother had cohabited with (other than participant’s father)					
None Vs. At least one	86 (54)	22 (48)	1.30 (0.64 – 2.53)	2.6	0.61
Mean number of persons that mother had cohabited with (other than participant’s father)	2.7 ± 0.3	4.0 ± 1.0	-------------	9.5**	**0.001**
Witness of physical fight among parents or guardian in the previous year					
Yes Vs. No	34 (22)	34 (70)	0.12 (0.06 – 0.25)	37.5	**<0.001**
Having been beaten by a parent or guardian to the point of serious bodily injury in the previous year*					
Yes Vs. No	85 (54)	23 (50)	1.2 (0.67 – 1.77)	0.2	0.89
Regular witness to use of alcohol or other substance by at least one parent or guardian in the last 1 year					
Yes Vs. No	58 (37)	12 (26)	1.64 (0.78 – 3.42)	1.7	0.18

*Before juvenile justice contact.

** Student *t*-test.

**Table 4:** Similarity and differences in the social circumstance before contact and the length of institutionalisation between the two categories of adolescents in institutional care in Nigeria

**Variable**	**Adolescents on Criminal Code (N = 158)**	**Cases of Child/Adolescent Neglect (N = 46)**	**OR (95% CI)**	**χ**^**2**^	**p**
Educational status*					
In School Vs. Dropped out of school	28 (18)	6 (13)	1.5 (1.2- 2.3)	10.1	**0.04**
Living on the streets as at the point of contact					
Yes Vs. No	105 (67)	25 (54)	1.8 (0.8 – 4.5)	4.3	0.06
Mean length of stay on the streets before contact (months)	22 ± 6	7 ± 2	------------	11.5**	**0.001**
Mean length of period of institutionalisation (months)	15 ± 7	18 ± 11	------------	7.6**	0.05

*Before juvenile justice contact.

** Student *t*-test.

### Neurological and anthropometric findings

The body mass index of the neglected adolescents was significantly lower and they were more likely to have neurological deficits and epilepsy compared with their ‘criminal code’ counterparts (Table 5). The neurological problems recorded among the neglected adolescents included slurred speech (n = 6), dyskinetic body movements (n = 4), reduced muscle bulk with hypertonia and hypereflexia (n = 5) and one participant with waddling gait.

**Table 5:** Similarity and differences in physical and mental status among the two categories of adolescents in institutional care in Nigeria

**Variable**	**Adolescents on Criminal Code (N = 158)**	**Cases of Child/Adolescent Neglect (N = 53)**	**OR (95% CI)**	**χ**^2^	**p**
**Anthropometric profile**					
Mean weight (Kg)	49.77 ± 5.41	34.93 ± 8.63	--------	6.3*	**0.01**
Mean height (Metres)	1.52 ± 0.21	1.43 ± 0.11	-------	1.6*	**0.04**
Mean body mass index (Kg/m^2^)	16.34 ± 0.33	15.85 ± 1.71	--------	7.7*	**0.03**
**Neurological Profile**					
Handedness (Right Vs. Left)	150 (95)	43 (93)#	1.7 (0.2 - 4.21)	0.3	0.88
Neurological deficits	1 (0.6)	12 (23)	0.03 (0.01- 0.09)	----	0.01**
Epilepsy	0 (0.0)	13 (25)	0.02 (0.01- 0.07)	-----	0.003**
**Common Mental Health Problems**					
Disruptive behaviour disorders	100 (63)	18 (39)#	2.6 (1.3 – 5.3)	10.8	**0.003**
Alcohol & substance abuse	96 (61)	5 (11)#	16.2 (5.6- 47.6)	23.8	**<0.001**
Multiple traumatic events+	43 (27.2)	12 (26) #	1.2 (0.8 – 1.9)	1.22	0. 43
Posttraumatic stress disorder	20 (13)	10 (22)#	0.52 (0.22- 1.29)	2.34	0.12

*Student *t*-test.

**Fisher’s exact statistic.

# n = 46.

+ Lifetime exposure to at least 3 different childhood traumatic events among the following: car or other accidents, being in a fire, witnessing a disaster, witnessing or being the victim of a violent crime, being confronted with traumatic news, sexual abuse.

### Common mental health problems

High lifetime prevalence rates of common mental health problems were recorded in the two groups of participants but the ‘criminal code’ group were twice more likely to have disruptive behaviour disorders and about 16 times more likely to have used or abused alcohol and substances compared with the neglected adolescents (Table 5).

## Discussion

Disruptive social changes in the society are the root cause of the difficult circumstances that many children of the world find themselves [38,39]. Social disruptions lead to a breakdown of structures of the society including the family unit. In resource-poor regions like most part of sub-Sahara Africa, central to the cause of social disruption is poverty and inequalities [5]. Poverty and inequalities destabilises the care environment of the child by robbing parents of the needed physical, emotional and financial resources for optimal childcare. Life of poverty and inequalities can also put severe strains on spousal relationships which can unsettle the stability of the family [40]. It has been reported that in the context of poor socio-economic circumstance, family instability is one of the root causes of delinquency and other socially deviant behaviours in children [41,42]. UNICEF also described the family as an essential element of the protective environment which ensures that children are shielded from all forms of abuse and neglect [6].

Though the concept of the ideal family unit may vary across cultures, the chances of optimal care and well-being of the average child is most guaranteed in a stable, consistent and harmonious unit formed by their married biological parents [43]. The two groups of adolescents in this study had a lot in common in terms of problematic indices of family structure, family consistency and family stability. This is in keeping with existing body of evidence that have established an enduring link between chequered family structure and dynamics and a higher risk of both juvenile delinquency and child abuse or neglect. Studies have shown that marital separation or single parenthood increases the risk of delinquent and criminal behaviours in children [44-46] while children from single-parent families and ‘broken homes’ are also known to be at higher risk of abuse and neglect [47,48]. Marital hostility or instability, which may manifest as frequent verbal conflicts or domestic violence has also been implicated as a risk factor for the duo of juvenile delinquency [49-51] and child neglect/abuse [52]. Similarly, considerably robust data have established a link between frequent changes in family structure (family transition) and delinquency [53-57] as well as child neglect [54,58].

Single-parent families, conflict-prone dual-parent families as well as families with rapidly changing composition and structure all have factors inherent in them that make it difficult for them to provide proper parenting and as such with higher risk of child neglect and delinquency among their children. For instance, in single-parent families, parental supervision is compromised by the fact that there is one less person to complement the supervision of children [59] while hostile family environment in unstable dual-families may create a distraction for optimal parenting. Family transitions on the other hand could compromise parenting quality through inconsistent discipline [60], parental loss of authority to exercise control, as well as parental preoccupation with the life changes that comes with family transition [61]. Poor parenting has been suggested to be one of the strongest predictors of delinquency [56] and standard contemporary definitions of child neglect [62] always includes an element of deficient parenting among the acts of omission that constitutes child neglect.

Another area of similarity that may suggest a common risk factor among the adolescents in this study irrespective of categorization includes certain aspects of their pre-contact social situation and lifetime mental health. For instance, more than half of both groups of adolescents had been living on the streets before juvenile justice contact. Leaving home to live on the streets could be a manifestation of the behavioral problems associated with juvenile delinquency [63]. Extant literature also suggests that running away from home to live on the streets could also be a child’s response to neglect [64,65]. In the context of sub-Sahara Africa, it has been argued that poverty-driven life of want, neglect, abuse and denial of the bliss of stable family environment has lead many children to the streets in search of life [10,66]. Whether being on the streets was as a result of neglect or delinquency or both, most street children run equal and continuous risk of juvenile justice contact. As far back as 1978, living or working on the streets had been reported as a common reason for juvenile justice contact in Nigeria [67]. Okunsanya in 2004 also reported that up to 15% of the total residents of remand and borstal facilities in Lagos Nigeria were brought-in after police raids on the streets [26]. In a recent survey of adolescents in the Ibadan Remand Home in Nigeria Bella et al. (2010) also reported that many of the residents in the Home had had some street-life experience irrespective of their reason for admission [17].

As a central common factor, problematic family background seems to be the linking factor that adversely changed the developmental trajectories of the two groups of adolescents in this study. This is because family instability and disruptions as seen in the two groups of adolescents in this study can trigger a cascade of events that terminates in juvenile justice contact. For instance, family instability can increase the risk of both child maltreatment [68] and conduct problems among children [69,70]. Living on the streets for children from unstable homes in sub-Sahara Africa is commonly a response to child maltreatment or an indicator of onset of conduct problems [13,63]. Once on the streets, children continue to run the risk of juvenile justice contact either as a status offender, due to involvement in crimes or involvement in substance use/abuse [26,71]. Though a cross-sectional study like this one can not establish causality, this study found all the factors needed to set up this cascade of events that terminates in juvenile justice contact among the two groups of adolescents. This include the combination of a high prevalence of indices of family instability on one hand, and other adverse factors like physical abuse, conduct disorder and experience of street life on the other hand.

On a different note, if the adolescents in this study share in common this much in terms of pre-contact primary support resources and social circumstance, what factors could have influenced their differential pathways (neglect or delinquency) to institutional care? The answer may lie in the fundamental differences in the pre-contact social situation and primary support resources of the two groups of adolescents in this study. Despite their stunning similarities, the two groups of adolescents have some distinct differences in terms of their family background, pre-contact social situation and their current anthropometric and lifetime mental health status. These differences may explain their differential pathways into institutional care and may have implications for specific prevention strategies and for service provision in-house. Though an element of abuse/neglect can be inferred in the life of both groups of adolescents in this study (e.g. street life, school drop-out, physical abuse etc.), the child-neglect group of adolescents had significantly worse indices in some neglect areas. For instance they had a higher risk of having witnessed domestic violence; they were more likely to have dropped out of school and had a significantly lower body mass index (BMI) compared with their counterparts on ‘criminal code’. These differences were despite the fact that the length of the mean period of incarceration among the two groups of adolescents was not significantly different. This observation may suggest that the two groups of adolescents in institutional care in Nigeria represents two points in a continuum of pathological social outcome of children, with the ‘child neglect’ pathway being the worst end of the continuum.

This assertion presupposes that the ‘criminal code’ group represents a pathway which resulted from a milder form of inadequate parenting and child neglect, while the ‘child-neglect’ group resulted from a more severe form of parental deficiency and neglect. The younger age, the worse indices of family transitions and the higher prevalence of physical disabilities among the child-neglect group in this study could all have put them at a higher risk of neglect and maltreatment. Studies have established an inverse relationship between the age of a child and the risk and severity of child maltreatment and neglect [72]. Younger children due to their earlier developmental status and higher need for care can be particularly vulnerable to maltreatment and neglect. With advancing age of a child, the pressures of parenting and the burden of childcare which may increase the tendency towards child maltreatment and neglect [73]; tend to reduce. In the same vein, if family transition becomes too rapid, the chances of coming in contact with abusive caregivers increase. Likewise, chronic disabling neurological disorders are additional risk factors for being abused or neglected [74,75]. Widespread misconceptions about the causation of childhood seizure disorders and neurological disorders in this region in particular have been cited as a major risk for neglect of children with such conditions [76]. These factors could have put the ‘child neglect’ group of adolescents in this study on a higher risk of worse forms of neglect and an institutional entry pathway under the neglect category.

Child neglect itself is a well documented precursor of delinquency [77,78]. This fact may suggest that adolescents with a more severe form of neglect will come in early contact with childcare services as a ‘neglect case’ while those with milder forms of neglect will progress further into ‘delinquency’ and will come in contact with childcare services much later. The observation that the ‘child neglect’ group in this study reported a lower mean number of months on the streets before juvenile justice or social-welfare contact compared with the ‘criminal code’ group supports this view. Another interesting observation which supports this view is the finding that more than half of the adolescents on ‘criminal code’ in this study were actually brought to the juvenile justice and social-welfare officers as the case may be, by their parent(s) or guardian(s) on the grounds that the adolescents were ‘beyond parental control’. This observation may suggest that being declared as ‘beyond parental control’ as an adolescent in Nigeria is a ‘milder’ or ‘formalized’ form of abandonment/neglect due to child behavioral problems and dwindling quality of primary support.

From the foregoing, it can be argued that the common factors that led to contact with juvenile justice and social welfare systems among two groups of adolescents in this study (irrespective of their categorisation) lies somewhere in the workings of their families. Family support programmes has been argued, and rightly so, to be one of the universal ways to stem the rising cases of adverse social outcomes for children generally in this region [79]. However, despite this effort, childcare can still be severely affected in deeply troubled families with attendant risks for children. In developed countries, children from families-at-risk can be put in foster families by the social welfare services. The foster families are given adequate funds to cater for the needs of such children including food, clothing, daily supervision, educational materials among others [80]. Though there is robust data that children in foster care experience a myriad of mental health problem that may affect the quality of their adulthood [81-83], the same, if not worse, has been reported among children and adolescents in institutional care [29,33]. Children in foster care are however given an opportunity to grow within a family setting which has been established long ago to be better than growing-up in institutions, from mental health perspectives [84]. Another possible advantage of the foster-care system is that children within this system are able to maintain contact with the community while in custodial care, which is likely to facilitate easy re-integration with own family and society. Nigeria and in deed most countries in sub-Sahara Africa are yet to evolve their foster care arrangements to formal systems and there is virtually no evidence of any research activity going on the feasibility of establishing such. This is ostensibly because of the low level of evolution of the social-welfare systems in this region which is currently focussed on keeping delinquent or offending children as well as neglected children off the streets and within confinement of institutions. This study therefore calls for an urgent research into the feasibility of establishing foster-care systems in Nigeria. A good approach will include research into perceived obstacles among social-welfare and juvenile justice officers, and willingness or reservations of families in the community to participate as destination-families in foster-care systems.

Furthermore, it was observed in this study that the ‘crimes’ committed by the adolescents on ‘criminal code’ were largely a declaration of inability of their parents to continue with their care ostensibly because of a combination of parental factors like social difficulties and child factors like disruptive behaviour disorders. Most of the other offences are either minor or status offences. Rather than the current practice in Nigeria, non-incarcerating methods of custodian care has been described as the ideal for status offenders as well as children in need of care and protection [85,86]. Modern non-incarcerating or semi-incarcerating facilities and diversion schemes in the community which cater for such children and adolescents include approved schools and the family foster care system [85,86]. This should be the way to go for Nigeria and her counterparts in sub-Sahara Africa.

It does appear from the findings of this study however that until the establishment non-incarcerating modes of child services and the full separation of the criminal and social welfare aspects of juvenile justice administration in this region, a common approach to the prevention and management of child neglect and delinquency still have some merit. Aside family interventions high rates of mental health problems in the two groups calls for the establishment of inclusion of mental health services generally and trauma services specifically in the multipurpose juvenile remand facilities scattered around the country. On the contrary, the significantly higher prevalence of disruptive behaviour disorders, substance use disorders and neurological disorders respectively among the ‘criminal code’ and child-neglect group suggests some specific needs. It may be imperative to emphasize behavioural modifications and therapy as a mental health service for the ‘criminal code’ adolescents and paediatric neurology and physical therapy services for the neglect group.

Until such time in Nigeria and indeed sub-Sahara Africa when the social-welfare and the criminal justice sections of child services are fully developed and separated, a reasonable management approach is to raise a local visiting team comprising of child and adolescent mental health experts, special education experts and paediatric neurologists and physical therapists within the vicinity of such institution. Such teams will provide needed services with the common and specific needs of the residents as highlighted in this study in mind. Part of the brief of such team could include some skill transfer to designated officials of the Home through direct trainings and seminars. The team could be sourced from secondary and tertiary health facilities in the vicinity of the juvenile home and could be funded by governments or donor agencies.

This is the first study in this region, to the best of author’s knowledge to explore the similarities and differences between the two groups of adolescent within Nigeria’s multipurpose childcare systems. A major limitation of this study is the fact that samples were drawn from only two institutions. This may limit the generalisation of the findings. The study would have also had more robust data if the participants took part in the study just before going into the systems. A large scale study involving point-of-entry samples drawn from at least one facility in the six geo-political zones in the country will yield a more representative and robust sample. The study also relied solely on the correctness of the information given by the adolescents as no effort was made to verify their claims about their family background. This was due to the non-availability of the huge resources that will be required to trace their families for confirmation.

## Conclusions

This study shows that the two broad groups of adolescents residing in the juvenile justice facilities studied had a lot in common in terms of pre-contact static and dynamic family structure; pre-contact social situation and current physical and mental status. It also provided a modest ground to infer that among children and adolescents in Nigeria, neglect and delinquency are different points of a spectrum. The study calls for urgent need to develop the non-incarcerating modes of childcare services in Nigeria and points the direction for future research.

## Competing interests

The author declares no competing interest.

## Author’s contributions

OA is the sole author.

## Pre-publication history

The pre-publication history for this paper can be accessed here:

http://www.biomedcentral.com/1471-2458/12/554/prepub

## References

[B1] Harper C Jones N McKay A Espey J Children in times of economic crisis: Past lessons, future policies 2009 Overseas Development Institute, UK

[B2] United Nations World Youth Report Juvenile Delinquency 2003 189 211 www.un.org/esa/socdev/unyin/documents/ch07.pdf

[B3] Nations U World Population Prospects: The 2008 revision 2008 Department of Economic and Social Affairs, Population Division

[B4] International Labour Organisation World Social Security Report 2010 – 2011 2011 Providing Coverage in Times of Crisis and Beyond, Geneva

[B5] Urban Management Programme Streetchildren and gangs in African cities: guidelines for local authorities http://www.unhabitat.org/downloads/docs/1901_41571_Streetchildren1.pdf

[B6] UNICEF The child in the family 2005 www.unicef.org/childfamily/index_24511.htm

[B7] Maru HM Kathuku DM Ndetei DM Psychiatry morbidity among children and young persons appearing in the Nairobi juvenile court Kenya East Afr Med J 2003 80 6 281 12953735

[B8] UNICEF Children in conflict with the Law in Uganda: Situation analysis 2000 UNICEF/Save the Children Group, UK

[B9] Ogundipe O Management of juvenile delinquency in Nigeria. Paper presented at the International Conference on Special Needs Offenders 2011 International Institute for Special Needs Offenders and Policy Research (Canada), Nairobi Kenya

[B10] Bwibo NO Onyango P Final Report of the Child Labour and Health Research 1987 University of Nairobi, Nairobi

[B11] Idemudia ES Personality and criminal outcomes of homeless youth in a Nigerian jail population: results of PDS and MAACL-H assessments J Child Adolesc Ment Health 2007 19 2 137 145 10.2989/17280580709486649 25865446

[B12] Obioha E Becoming a Street Child in Poverty Ridden Society: A Descriptive Case of Kaduna Metropolis Nigeria Journal of Soc Sc 2009 19 1 41 49

[B13] Assiago Youth and Crime in Nairobi: Some Exploratory Issues 2002 UN Habitat Safer Cities Programme, Nairobi

[B14] Petty C Brown M(eds): Justice for Children: Challenges for Policy and Practice in Sub-Saharan Africa 1998 Save the Children, June

[B15] Sam A Child justice in Africa 2007 PREDA Foundation. Olongapo city, Philippines

[B16] Okumu T Mental Health and Substance abuse problems among Juvenile Offenders at Getathuru children's reception and Rehabilitation centre 2007 Postgraduate project submitted to University of Nairobi, Nairobi –Kenya

[B17] Bella T Atilola O Omigbodun O Children within the Juvenile Justice System in Nigeria: Psychopathology and Psychosocial Needs Annals of Ibadan Pg Med 2010 8 1 34 39 10.4314/aipm.v8i1.80344 PMC4138769 25161473

[B18] Ajiboye PO Yusuff AD Issa BA Adegunloye OA Buhari ON Current and lifetime prevalence of mental disorders in a juvenile Borstal Institution in Nigeria Research Journal Medical Science 2009 3 1 26 30

[B19] Ononye F Morakinyo O Drug abuse, psychopathology and juvenile delinquency in south – west Nigeria Journal of forensic psychiatry and psychology 1994 5 3 527 537

[B20] Szilagyi M The pediatrician and the child in foster care Pediatr Rev 1998 19 39 10.1542/pir.19-2-39 9473940

[B21] Mather M Adoption: a forgotten paediatric speciality Arch Dis Child 1999 81 492 10.1136/adc.81.6.492 10569965 PMC1718157

[B22] The world fact book 2011 www.cia.gov/library/publications/the-world-factbook

[B23] UNDP International Human Development Indicators. Nigeria country profile http://hdrstats.undp.org/en/countries/profiles/NGA

[B24] Abdulmalik J Omigbodun O Beida O Adedokun B Psychoactive substance use among children in informal religious schools (Almajiris) in northern Nigeria Ment Health, Rel & Culture 2009 12 6 527 542 10.1080/13674670902832813

[B25] Aransiola JO Bamiwuye O Akinyemi AI Ikuteyijo LO Proliferation of Street Children in Nigeria: Issues and Challenges J Soc Work 2009 9 4 371 385 10.1177/1468017309342539

[B26] Okunsanya A Owasanoye B, Wernham M Remand Facilities, Approved Schools and the Post Trial Processing of Children Street children and juvenile justice system in Lagos State of Nigeria Human Development Initiatives, Nigeria 47 61 Chapter 5

[B27] Azar S Parenting and child maltreatment 2002 Lawrence Erlbaum Associates, 361 88

[B28] Tolnay S Crowder K Regional origin and family stability in northern cities: The role of context Am Sociol Rev 1999 64 1 97 112 10.2307/2657280

[B29] Teplin L Abram K McClelland G Psychiatric disorders in youth in juvenile detention Arch Gen Psychiatry 2002 59 1133 1143 10.1001/archpsyc.59.12.1133 12470130 PMC2861992

[B30] Fazel S Doll H Långström N Mental disorders among adolescents in juvenile detention and correctional facilities: A systematic review and met regression analysis of 25 surveys J of Ame Acad of Child & Adolesc Psychiatry 2008 47 9 1010 1019 10.1097/CHI.ObO13e31817eecf3 18664994

[B31] Adegunloye OA Yusuf AD Ajiboye PO Issa BA Buhari ON Prevalence and Correlates of Distruptive Behaviour Disorders in Youths in a Juvenile Borstal Institution Nig J of Psychiatry 2010 8 3 12 17

[B32] Wasserman GA Miller L Pinner E Jaramillo BS Parenting predictors of early conduct problems in urban, high-risk boys J of Am Acad of Child & Adolesc Psychiatry 1996 35 1227 1236 10.1097/00004583-199609000-00020 8824066

[B33] Abram KM Teplin LA Charles DR Posttraumatic Stress Disorder and Trauma in Youth in Juvenile Detention Arch Gen Psychiatry 2004 61 403 410 10.1001/archpsyc.61.4.403 15066899 PMC2861915

[B34] Carrion VG Steiner H Trauma and dissociation in delinquent adolescents J Am Acad Child Adolesc Psychiatry 2000 39 353 359 10.1097/00004583-200003000-00018 10714056

[B35] World Health Organization Adolescent Friendly Health Services: An agenda for change 2002 WHO, Geneva 13

[B36] Costello A Developments in child psychiatric epidemiology J Am Acad Child Adolesc Psychiatry 1989 28 836 841 10.1097/00004583-198911000-00004 2681135

[B37] Lavigne JV Gibbons RD Christoffel KK Arend R Prevalence Rates and Correlates of Psychiatric Disorders among Preschool Children J Am Acad Child Adolesc Psychiatry 1996 35 2 204 14 10.1097/00004583-199602000-00014 8720630

[B38] Redman CL Jones NS The Environmental, Social, and Health Dimensions of Urban Expansion. Produced for a Population-Environment Research Network cyber-seminar 2004 www.populationenvironmentresearch.org

[B39] UNICEF Children in an Urban World. The state of the World’s Children 2012 United Nations Publications, NY USA

[B40] Conger KJ Rueter MA Conger RD Crockett LJ, Silbereisen RJ The role of economic pressure in the lives of parents and their adolescents: The Family Stress Model Negotiating adolescence in times of social change 2000 Cambridge University Press, Cambridge 201 223

[B41] Before Head Start: income and ethnicity, family characteristics, child care experiences and child development Early Educ Dev 2001 12 545 576 10.1207/s15566935eed1204_4

[B42] Yoshikawa H Long-Term Effects of Early Childhood Programs on Social Outcomes and Delinquency The Future of Children: Long-Term Outcomes of Early Childhood Programs 1995 5 (3) 51 75 8835514

[B43] Moore KA Jekielek SM Emig C Marriage from a child's perspective: How does family structure affect children, and what can we do about it 2002 Child Trends Research Brief, Washington, DC

[B44] Manning WD Lamb KA Adolescent Well-Being in Cohabiting Married, and Single-Parent Families, J of Mar and Family 2003 65 876 893

[B45] Wright KN Wright KE Family Life, Delinquency & Crime: A Policymakers Guide. Research Summary 1994 OJJDP, Washington DC 4 21

[B46] The case for two-parent family Part II National Observer 2002 53 49 58

[B47] Child Abuse and Violence. In Single-Parent Families: Parent Absence and Economic Deprivation Am J of Orthopsychiatry 1989 59 4 492–501 2817087 10.1111/j.1939-0025.1989.tb02738.x

[B48] Goldman J Salus M Wolcott D Kennedy K A Coordinated Response to Child Abuse and Neglect: The Foundation for Practice. Child Abuse and Neglect User Manual Series 2003 Department of Health and Human Services, Office on Child Abuse and Neglect, Washington, D.C.: U.S

[B49] Cummings EM Davies P Children and Marital Conflict 1994 Guilford, New York

[B50] Gorman-Smith D Tolan P Sheidow AJ Henry DB Partner Violence and Street Violence among Urban Adolescents: Do the Same Family Factors Relate J of Res on Adolescence 2011 11 273 95

[B51] Webster-Stratton C The Incredible Years 2003 Umbrella Press, Toronto

[B52] Cawson P Child Maltreatment in the Family: The experience of a national sample of young people 2002 National Society for the Prevention of Cruelty to Children, London

[B53] Adam EK Chase-Lansdale L Home Sweet Home(s): Parental Separations, Residential Moves, and Adjustment Problems in Low-Income Adolescent Girls Dev Psychology 2002 38 5 792 805 12220056

[B54] Brown SL Family Structure Transitions and Adolescent Well-being Demography 2006 43 3 447 461 10.1353/dem.2006.0021 17051822

[B55] Juby H Farington DP Disentangling the link between disrupted families and delinquency Br J of Criminology 2001 41 22 40 10.1093/bjc/41.1.22

[B56] Hawkins DF Laub JH Lauritsen JL Loeber R, Farrington DP Serious and Violent Juvenile Offenders Risk Factors and Successful Interventions 1998 Sage Publications, Inc, Thousand Oaks, CA

[B57] Farrington D Maguire M, Morgan R, Reiner R Developmental Criminology and Risk-Focused Prevention The Oxford Handbook of Criminology, Third Edition (657–701) 2002 University of Oxford Press, Oxford

[B58] Waters E Cummings M A Secure Base from Which to Explore Close Relationships Child Development 2000 71 164 172 10.1111/1467-8624.00130 10836570

[B59] Wright KN Wright KE Family Life, Delinquency & Crime: A Policymakers Guide 1994 Research Summary, Washington DC 4 21

[B60] Thornberry TP Smith AS Rivera C Huizinga D 1999 Family Disruption and Delinquency. Office of Juvenile Justice and Delinquency Prevention Washington DC, Stouthamer-Loeber M www.ncjrs.gov/pdffiles1/ojjdp/178285.pdf

[B61] Hetherington EM Social Support and the Adjustment of Children in Divorced and Remarried Families Childhood 2003 10 2 217 236 10.1177/0907568203010002007

[B62] Definitions of child abuse and neglect 2011 Department of Health and Human Services, Children's Bureau, Washington, DC: U.S

[B63] Owasanoye B Owasanoye B, Wernham M Street Children and the Juvenile Justice System in Lagos State Street children and juvenile justice system in Lagos State of Nigeria 2004 Human Development Initiatives, Nigeria 2 9 Chapter 2

[B64] Rees G Siakeu J Thrown Away: The Experiences of Young People Forced to Leave Home 2004 The Children’s Society, London

[B65] Smeaton E Living on the Edge: The Experiences of Detached Young Runaways 2005 The Children’s Society, London

[B66] Ebigbo P Street Children: The Core of Child Abuse and Neglect in Nigeria Children, Youth and Environments 2003 13 1 22 31

[B67] Odiase GI Ebie JC A report of juvenile delinquency over a two year period in benin city Ment Health Soc 1978 5 5–6 325 9 555504

[B68] Shepard M Raschick M How Child Welfare Workers Assess and Intervene around Issues of Domestic Violence Child Maltreatment 1999 4 148 156 10.1177/1077559599004002007

[B69] Mash JE Wolfe DA Abnormal child psychology 2007 Thomson: Wadsworth, Belmont, CA

[B70] Matthews J Family Risk Factors and Conduct Disorder among Committed Male and Female Juveniles in Barbados Caribbean Journal of Psychology 2011 4 1 5 16

[B71] Omiyinka FO Social networks and livelihood of street children in Ibadan, Nigeria International Journal of Sociology and Anthropology 2009 1 5 82 89

[B72] Administration on Children, Youth and Families: Child Maltreatment 2004 US Government Printing Office, Washington, DC

[B73] Burke J Chandy J Dannerbeck A Watt JW The parental environment cluster model of child neglect: An integrative conceptual model Child Welfare 1998 77 4 389 9666551

[B74] Spencer N Devereux E Wallace A Sundrum R Disabling conditions and registration for child abuse and neglect: a population based study Pediatrics 2005 116 609 613 10.1542/peds.2004-1882 16140700

[B75] Sullivan PM Knutson JF Maltreatment and disabilities: a population-based epidemiological study Child Abuse and Neglect 2000 24 10 1257 1273 10.1016/S0145-2134(00)00190-3 11075694

[B76] Abasiubong F Obembe A Ekpo E The Opinion of Mothers to Mental Retardation in Lagos Nigeria. Nig J of Psychiatry 2008 6 2 2 2

[B77] Malinosky-rummell R Hansen DJ Long term consequences of childhood physical abuse Psychtr Bull 1993 114 68 79 10.1037/0033-2909.114.1.68 8346329

[B78] Maxfield M Widom C The cycle of violence: Revisited 6 years after Arch of Paed & adol Medicine 1996 150 4 390 10.1001/archpedi.1996.02170290056009 8634734

[B79] Atilola O Can family interventions be a strategy for curtailing delinquency and neglect in Nigeria? Evidence from adolescents in custodial care African Journal for Psychological Studies of Social Issues 2012 15 1 218 237

[B80] Federal Definition of Foster Care and Related Terms. Title 45, Volume 4, Parts 1200, Sec. 1355.20 2000 GPO, U.S

[B81] Bruskas D Children in Foster Care: A Vulnerable Population at Risk J of Child and Adolescent Psychiatric Nursing 2008 21 2 70 77 10.1111/j.1744-6171.2008.00134.x 18429837

[B82] Garland AF Hough RL McCabe KM Yeh M Prevalence of psychiatric disorders in youths across five sectors of care Am Acad Child Adolesc Psychiatry 2001 40 409 418 10.1097/00004583-200104000-00009 11314566

[B83] McMillen JC Zima BT Scott LD Auslander WF, et al: Prevalence of psychiatric disorders among older youths in the foster care system Am Acad Child Adolesc Psychiatry 2005 1 88 95 10.1097/01.chi.0000145806.24274.d2 15608548

[B84] Bowlby J Maternal care and mental health Bull of the WHO 1951 3 335 534 PMC2554008 14821768

[B85] Crowell P Community based corrections 2001 Wardsworth Publishing Company, Belmont, CA

[B86] Arbuckle M Jones HC Child & adolescent mental health: interdisciplinary systems of care 2006 Bartlett Publishers Inc, Burlington, MA 233 234

